# Production of Metastases by a Primary Tumour Irradiated under Aerobic and Anaerobic Conditions in Vivo

**DOI:** 10.1038/bjc.1972.53

**Published:** 1972-10

**Authors:** H. A. S. van den Brenk, V. Moore, C. Sharpington, C. Orton

## Abstract

**Images:**


*Br. J. Cancer* (1972) **26**, 402

### PRODUCTION OF METASTASES BY A PRIMARY TUMOUR IRRADIATED UNDER AEROBIC AND ANAEROBIC CONDITIONS *IN VIVO*

H. A. S. VAN DEN BRENK, V. MOORE, C. SHARPINGTON AND C. ORTON

*From the Richard Dimbleby Research Laboratory, St. Thomas' Hospital, London, S.E.1*

Received 3 March 1972. Accepted 6 June 1972

**Summary.—The effect of local irradiation of a rapidly metastasizing sarcoma in the leg of the rat was measured in terms of (a) regression of the primary tumour and (b) growth of metastases produced in lymph nodes and lungs, by dissemination occurring after irradiation of the primary tumour. These effects on rats which had been irradiated while breathing air were compared with rats breathing 10% $O_2$/90% $N_2$ in which a tourniquet had been applied proximal to the tumour to arrest blood flow during irradiation. Tourniquet anoxia increased radioresistance of growth of primary tumour by (OER) factors of 2·9–3·3. Corresponding factors for inhibition of growth of metastases in abdominal lymph nodes, and for the reduction in incidence of lung metastases produced by single tumour cells, were 2·7 and 2·4 respectively. These results suggest that this tumour was radiobiologically well oxygenated when it was irradiated in a poorly vascularized stage of growth where tumour necrosis had developed.**

It has been shown that the Y-P388 variant of Yoshida sarcoma, inoculated intramuscularly in the leg of rats previously given a sublethal dose of whole body irradiation, metastasizes regularly and rapidly to lymph nodes and lungs. The growth of the metastases is quantitatively related to the growth of the primary inoculum (van den Brenk, Moore and Sharpington, 1971). Further experiments have shown that local irradiation of the primary tumour causes radiation dose-dependent reduction in dissemination and growth of metastases occurring after irradiation in both lymph nodes and lungs (van den Brenk and Sharpington, 1972). The present paper describes experiments in which the primary tumour was irradiated locally, either under aerated conditions (the rats breathing air during irradiation) or under anoxic conditions (when a tourniquet was applied to the leg proximal to the primary tumour, thus obstructing tumour blood supply). Growth of metastases after irradiation was measured.

### MATERIALS AND METHODS

The methods adopted to passage and inoculate the Y-P388 sarcoma, the SPF rats used for tumour growth, and the whole body and local irradiation techniques have been described previously (van den Brenk *et al.*, 1971; van den Brenk and Sharpington, 1972). To occlude blood flow to the tumour growing in muscle of the leg of the rat, a tourniquet was applied proximal to the tumour as described previously in mice (van den Brenk, Elliott and Hutchings, 1962) and in humans (van den Brenk *et al.*, 1963). The rats were anaesthetized with pentobarbitone sodium, given intraperitoneally, and the tourniquet was applied for 15–20 minutes before as well as during irradiation. During occlusion by tourniquet, the rats breathed a nitrogen with 10% oxygen gas mixture through modified Gaddum masks to reduce oxygenation of the blood and increase tumour anoxia. After irradiation, the tourniquet was released immediately, restoring the blood flow in the limb. This caused a reactive hyperaemia in all rats. No rats died but approximately one third of the tourniquet treated rats (whether irradiated or control) developed a tourniquet palsy. No ulceration appeared at the site of tourniquet application. In rats irradiated under aerated conditions, the elastic tourniquet was applied loosely without pressure in order to preserve the same radiation scatter factor and uniformity of dose. These rats breathed air before and during irradiation.

The limb was exposed to a single dose of local irradiation (750–4000 rad) 48 hours after inoculation of $10^6$ Y-P388 cells suspended in 0·1 ml Tyrode solution (pH 7·6) into the distal third of the right gastrocnemius muscle. The radiation factors were 212 kV, 15 mA, HVL 1 mm Cu, dose rate 570 rad $min^{-1}$; twin opposed x-ray beams, described previously, were used. Four days after irradiation of the tumour, the size of the primary tumour was measured by the semi-quantitative method described previously. On the fifth day post irradiation the rat was killed and the primary tumour (Pr), ipsilateral popliteal (crural) lymph nodes (CN), pelvic (lower abdominal) lymph nodes (PN), upper abdominal lymph nodes (UAN), and the spleen and thymus were removed and weighed. The lungs were also removed and weighed and the number of tumour colonies present on the pleural surfaces of both lungs was counted.

A group of 6 female 6-week old rats which had received the same treatment was used to measure tumour growth. Each rat was exposed to 570 rad whole body irradiation ($^{60}Co$ $\gamma$ rays) 24 hours preceding inoculation to suppress immunological reactions to this allogeneic tumour. This treatment was found to reduce " take " and growth of tumour in muscle of 50% of rats inoculated ($ED_{50}$ value) from $\sim 5 \times 10^3$ to $<10$ Y-P388 cells.

*Tumour vasculature and response to radiation.*—A separate group of 6 female 6-week old rats was used to examine the blood supply of this tumour. Under pentobarbitone anaesthesia, a laparotomy was performed on each 6-week old rat, the stomach delivered into the wound and $\sim 10^5$ Y-P388 cells suspended in 0·01 ml Tyrode solution were injected with a microsyringe beneath the peritoneal covering of the anterior stomach fundus. The tumour was allowed to grow for 3–4 days and then the rat was exsanguinated under anaesthesia and the circulation perfused with warm normal saline containing a vasodilator (0·5% sodium nitrate) injected intracardially. This perfusion was followed by a further perfusion with warm india ink–gelatine mixture which impregnated the vasculature as described previously (Jamieson and van den Brenk, 1963). The stomach was removed rapidly, cooled in ice cold saline, opened and washed, pinned out on a square of polystyrene and transferred to 0·2% acetic acid at 4 °C, in which it remained overnight to facilitate removal of the mucosal layer. The tumour with surrounding stomach wall was fixed in neutral formalin, dehydrated, cleared and mounted in epoxy resin as an *en face* preparation for study of the tumour vasculature. Similar preparations of stomach tumours were made in rats which had been reanaesthetized when the tumours had grown to 3–8 mm in diameter, the laparotomy being then reopened and the stomach exposed, so that the tumour could be treated with a single dose (1500 rad) of x-radiation. These rats were killed 3–5 days after irradiation, after the vasculature had been impregnated with india ink as before. Similarly, ink-impregnated preparations were made of leg tumours, lymph nodes and lung metastases at various stages of tumour growth and also after irradiation. These tissues were fixed, embedded in paraffin and then 10–20 $\mu$ thick sections were prepared and counterstained with light green for microscopic examination of the vasculature.

*Cell viability following exposure to anoxia.*—Aliquots of freshly harvested Y-P388 ascites fluid were diluted with Tyrode solution ($\sim 10^6$ cells per ml) and gassed continuously for 1 hour at room temperature with either 5% $CO_2$/95% air or industrial nitrogen (specified by the manufacturers to contain $<10$ parts $10^6$ $O_2$). A further aliquot was incubated for one hour at 37 °C after adding NaCN ($10^{-6}$ mol/litre final concentration). After treatment, the suspensions were diluted with ice cold Tyrode to give $10^3$ cells per ml, and 0·5 ml ($5 \times 10^2$ cells) was inoculated intravenously into each rat. Groups of 4-week old weanling rats which had been given 570 rad whole body irradiation 2 hours previously were inoculated. These rats were killed on the seventh day and the number of macroscopic tumour colonies present on the surfaces of lungs and kidneys were counted to

(a)

(b)

(c)

FIG. 1.—(a) Five-day old tumour produced by $10^5$ Y-P388 cells inoculated into anterior wall of fundus of stomach; dark appearance of tumour is due to diffuse haemorrhage. There is displacement, crowding and destruction of stomach vasculature by tumour growth ( × 10). (b) Destruction of the vasculature of the muscular coat of stomach at the periphery of the tumour ( × 80). (c) Residual tumour (measuring approximately 2 mm in diameter) present in wall of stomach 5 days after exposure to 1500 rad x-rays when the tumour measured 8 mm in diameter; shows regression of tumour (and of haemorrhage) and a restoration of vascular patterns in stomach wall which has largely been due to vasculature previously displaced by tumour growth returning to a more normal position. These vessels appear somewhat more dilated after irradiation and regression of tumour ( × 10).

(All illustrations are of *en face* preparations of stomach after impregnation of the vasculature of rat with india ink–gelatine suspension).

measure cell survival in terms of colony-forming ability.

### RESULTS

### *Tumour vasculature*

The Y-P388 sarcoma is a rapidly growing tumour which destroys the normal tissues it infiltrates, including the blood vasculature. Possibly due to rapidity of tumour growth, little or no true new capillary growth (angiogenesis) appears to be stimulated—angiogenesis which would be required to provide the tumour with an adequate blood supply (Fig. 1). Tumour growth depends largely on pre-existing vascular networks for nutrition, but these are progressively destroyed and displaced by the tumour, so that most of the tumour develops into an essentially " avascular " structure and only a very thin ($\sim$1 mm thick) zone at the periphery contains patent blood vessels. Destruction of blood vessels causes marked haemorrhage into the tumour so that all growing primary and secondary deposits become blood red in colour. Heparinization of the animal, followed by exsanguination and prolonged perfusion of the vasculature with saline containing vasodilators to remove circulating blood, fails to remove this blood from the tumour. Histological examination of sections of tumour show that free " lakes " and extravasations of blood, not enclosed by endothelium, are present. These findings suggest that most blood present in solid Y-P388 tumour is non-circulating and stagnant. The avascular, haemorrhagic state of tumour growth could be seen 24 hours after inoculation. At this early stage of growth the inoculated cells were actively proliferating and infiltrating host tissues and giving rise to metastases, since amputation of the limb did not prevent formation of metastases in lymph nodes and lungs (van den Brenk *et al.*, 1971). Vascular damage and haemorrhage had occurred widely at 48 hours after injection of the leg muscle with $10^6$ cells when the tumours were irradiated.

The vasculature which remained patent at the periphery of the growing tumour showed some vasodilation. Tumour expansion due to its growth also caused displacement and " crowding " of the vasculature at the periphery. This needs to be distinguished from new growth of blood vessels (angiogenesis). More detailed microscopic examination of the peripheral vasculature at different depths in the tumour, where it had infiltrated the various (serous, muscular and mucosal) layers of the stomach wall and elsewhere, clearly showed general vascular destruction and there was no clear evidence of angiogenesis stimulated by tumour growth (Fig. 1b).

Exposure of the tumour to a higher (1500 rad) single dose of x-radiation caused rapid reduction in tumour dimensions, with resorption of tumour tissue. This allowed the previously displaced vasculature at the periphery to close in and resume more normal patterns of density and form (Fig. 1c). This change appeared to be largely passive; active vascularization of regressing tumour tissue was not obvious and, when present, angiogenesis was organizational in a reparative sense and consisted of granulation tissue foci growing into necrotic tumour. Inhibition of the growth of the tumour by irradiation caused cessation of haemorrhage and a rapid and progressive disappearance of accumulated blood and its products which had collected during tumour growth before irradiation.

*Aerobic and anaerobic radiation dose-responses*

(a) *Primary tumour* (*Pr*).—The primary tumour in leg muscle at 48 hours after inoculating $10^6$ tumour cells was more radiosensitive if irradiated " in air " than under anoxic conditions produced by tourniquet occlusion (Fig. 2). Measurements of palpable tumour size made 4 days after irradiation, and of weight of primary tumour when rats were sacrificed 5 days post-irradiation, gave similarly shaped dose-effect curves, and a similar decrease in radiosensitivity when the tourniquet had been applied to arrest blood flow during irradiation

(b) *Ipsilateral crural lymph node metastases* (*CN*).—These were included with Pr in the irradiation field distal to the tourniquet and showed an " oxygen effect " with respect to radiosensitivity similar to that of the primary tumour.

(c) *Abdominal lymph node metastases* (*PN*, *UAN*).—The metastases in lower abdomino-pelvic (PN) and upper abdominal (UAN) groups of lymph nodes result from cumulative growth of tumour produced by dissemination from both Pr and CN which occurs (a) in the first 48 hours after inoculation, *i.e.* before local irradiation of the leg and (b) after irradiation. This tumour spreads rapidly so that considerable dissemination occurs within 24–48 hours post-inoculation (van den Brenk and Sharpington, 1972). PN and UAN were shielded from exposure to irradiation so that dosage given to the leg did not affect cumulative growth of meta-

FIG. 2.—Dose-effect relationships for local irradiation (single doses) of primary tumour in leg of rat under ambient conditions of breathing air (closed symbols) and when the leg was made anoxic during irradiation by tourniquet occlusion (open symbols); Pr, primary tumour; CN, ipsilateral crural lymph nodes; PN, pelvic nodes; UAN, upper abdominal nodes.

stases in these nodes directly. Consequently, the dose-response curves showed 2 components: an initial decrease due to a dose-dependent reduction in dissemination occurring after irradiation, and a flattened region at higher doses which represents growth of metastases from dissemination that had already occurred at the time of irradiation. These dose-effect curves are modified in shape as a result of the action of growth stimulting substances (GSS) which are released by the irradiated primary tumour, as reported previously (van den Brenk and Sharpington, 1972). Greater amounts of GSS are liberated by higher doses of irradiation and can stimulate increased growth of metastases when little viable Pr remains to "capture" GSS locally. This is seen particularly for smaller, less advanced metastases, as in the present experiment for UAN (and lung metastases) when single doses of 4000 rad were given to reduce Pr growth and caused a secondary, upward curvature of the flat portion of the dose-response curves. Reductions in growth of PN and UAN metastases were dose-dependent for 0–2000 rad to Pr and were greater for irradiation of the leg under aerobic (" in air ") conditions. Therefore, the expected oxygen effect for tumour (and tissue) radiosensitivity *i.e.* greater radiosensitivity under aerated conditions, was manifested not only by the effect of radiation in inhibiting local growth of tumour, but also by the number of viable cells which exfoliated and disseminated to unirradiated lymph nodes after irradiation, to grow and produce metastases (see below and Fig. 3 for oxygen effect values).

(d) *Lung metastases.*—Reductions produced by irradiation of the tumour-bearing leg in the numbers of cells which disseminated to the lungs and produced single colonies followed the same pattern as growth of PN and UAN lymph node metastases, *i.e.* residual growth of metastases due to dissemination *before* Pr irradiation, stimulation of growth of lung metastases by GSS manifested at high dosage (4000 rad) to Pr and a dose-dependent reduction in dissemination showing dependence on the "oxygen effect " for spread occurring *after* irradiation. The effects of Pr irradiation on growth of lung metastases were reflected also by measurements made of lung weight, increase in lung weight being related to number (and size) of lung metastases.

*Oxygen-effect factors* (*OER*)

These have been estimated by normalizing the data in Fig. 2 and calculating the radiation dose required to reduce growth of tumour in the various sites by 50% (Fig. 3). The values obtained for OER were 2·9 and 3·3 for radiation effects on Pr measured on the sixth and seventh day of tumour growth respectively, 2·7 for combined weight (Po) of PN and UAN metastases and 2·4 for lung metastases.

*Susceptibility of Y-P*388 *cells to anoxia*

When tumour cells had been gassed continuously at room temperature with $N_2$ for 1 hour *in vitro*, and assayed for colony formation in lungs and kidneys by intravenous inoculation of immunologically suppressed rats, their viability was not decreased (Table I). Neither this treatment, nor exposure of the cells for the same length of time to NaCN, caused significant changes in survival and clonogenicity *in vivo*. Previous experiments had shown that this tumour was also resistant *in vitro* at 37 °C to other metabolic inhibitors (iodoacetate and N-ethyl maleimide) in high concentrations (van den Brenk *et al.*, 1971) and to anaerobic conditions at 37°C of similar duration. Further experiments have shown that incubation of unirradiated freshly harvested tumour ascites fluid ($1·5–2·0 \times 10^8$ cells/ml) at 37 °C for 4–8 hours in stoppered tubes allowed more than 50% of cells to retain proliferative integrity, based on intravenous inoculation and lung colony assays *in vivo*.

### DISCUSSION

The results have shown that early growth of this tumour in muscle takes

FIG. 3.—Dose-effect data in Fig. 2 normalized for effect on Pr measured clinically 6 days after inoculation ($C_6$), at necropsy as tumour weight on the seventh day ($W_7$), and for effect on combined weight (Po) of pelvic and upper abdominal lymph node metastases. Oxygen effect ratios calculated for 50% reductions in growth are shown as figures inserted on interrupted lines.

TABLE I.—*Incidence of Tumour Colonies Produced in Lungs and Kidneys 7 Days After Intravenous Inoculation of 4-week Old Female Rats With* 5 × 10² *Y-P*388 *Cells*

| | Number of tumour colonies | |
|---|---|---|
| Group | Lungs | Kidneys |
| A . . . | 42±13 | 2·8±0·8 |
| B ($N_2$) . . | 34±8 | 1·6±0·5 |
| C (NaCN) . | 51±13 | 1·6±0·9 |

Immediately preceding inoculation the tumour cells were suspended in Tyrode solution (10⁵ cells/ml) at 21 °C and treated for 60 minutes as follows: Group A, gassed with 5% $CO_2$/95% air; Group B, gassed with $N_2$ (containing < 10 p.p.m. $O_2$); Group C, gassed with 5% $CO_2$/95% air in the presence of $10^{-6}$ mol/litre NaCN. All rats received 570 rad WBI 24 hours preceding inoculation; 5 rats per group.

place under essentially avascular conditions. A stimulation of *new blood vessel growth* (angiogenesis) in support of growth of tumour has not been seen at any stage. Thus, the tumour depends on the existing capillary network it infiltrates for nutrition, and since this network is quickly destroyed and displaced by the rapidly enlarging tumour, this situation may be considered conducive to an early and progressive development of tumour anoxia. The most rapid form of angiogenesis known to occur in mammalian tissues is in wound healing and repair. Under optimal conditions reparative angiogenesis occurs at a mean rate not exceeding 0·3 mm per day, and is delayed for at least 2–3 days after injury, during which time demolition takes place and the remaining vasculature is reconstructed as a prelude to active angiogenesis (Clark and Clark, 1932; Needham, 1952; van den Brenk, 1956; Cliff, 1965; and Florey, 1970). These conditions would seem to preclude the likelihood that a rapidly growing tumour such as Y-P388 could become vascularized within the first 48 hours by regenerative growth of new blood vessels, particularly since the tumour as it grows destroys and displaces the vasculature from the onset. Furthermore, after the stomach wall had been irradiated with 4000 rad, an inoculum of Y-P388 tumour grew rapidly and produced the same vascular disturbances seen in unirradiated tissue (unpublished data; see also Fig. 1a). This dose of irradiation has been shown to inhibit new growth of blood vessels entirely (van den Brenk, 1959) and to reduce proliferative integrity of oxygenated mammalian cells to insignificant levels.

Invasion of the host vasculature by tumour causes profuse haemorrhage into the tumour. *In vitro* experiments have shown that cells of this tumour can withstand prolonged exposure to anoxia and inhibition of metabolic activities, without resulting in significant loss of viability and clonogenicity. Despite these pathophysiological characteristics of growth, the 48-hour old solid tumour in muscle has been shown to be well oxygenated from a radiobiological standpoint, since complete occlusion of blood supply to the primary tumour caused a considerable increase in radioresistance with an oxygen effect factor (OER) in excess of 2·5, *i.e.* a greater than 2·5-fold increase in radiation dose administered under anoxic conditions was required to cause the same degree of inhibition of growth (measured in terms of weight of tumour) as was produced by irradiation when the circulation to the leg of an animal breathing air under ambient conditions was not arrested. Such measurements of radiation effect, in terms of decrease in tumour volume or weight, depend not only on cellular depopulation but also on other radiation sensitive (but not necessarily lethal) cellular events, such as mitotic delay and effects on tumour bed (stroma), and therefore may not be a true reflection of cell survival. However, growth of metastases in unirradiated tissues produced by exfoliation and dissemination of tumour cells from the irradiated tumour is related much more closely to replicative integrity, and in the lungs macroscopic colonies produced by the deposition of single tumour cells represent clonogenicity of the tumour *in vivo*. Estimates of OER based on exfoliation of cells from the irradiated primary and their growth as metastases were similar for lymph node and lung metastases, and support the view that the growing 48-hour old tumour in muscle is well oxygenated with respect to radiosensitivity in the anaesthetized rat breathing air at atmospheric pressure, despite clear-cut evidence of vascular insufficiency at this stage. An explanation for this paradox may be that viable tumour cells occur only in a narrow peripheral zone in closer proximity to intact host blood vessels, and that the latter maintain oxygen tensions in this peripheral zone at the 5–10 mm Hg $pO_2$ level required for near maximal radiosensitivity in accordance with the relationship of Alper and Howard-Flanders (1956). However, an abundance of apparently viable tumour cells, including those which label with $^3H$-thymidine and show mitoses, were seen in more central avascular regions, not only in 48-hour old but in much larger tumours. Y-P388 tumour grew and metastasized too rapidly for similar measurements of radiosensitivity to be made for larger, older tumours. However, preliminary experiments using a 5-day old tumour in the rat, which metastasizes much less rapidly and regularly, have also given relatively high OER values ($\sim$2·2) for irradiation under ambient conditions when the primary tumour was 1 cm or more in diameter, showed widespread necrosis and had a vascular structure accompanied by marked haemorrhage similar to Y-P388 tumour.

It is suggested that in a haemorrhagic tumour, diffusion of oxygen from the vascularized periphery to deeper avascular regions may be facilitated by pools of free blood and blood pigments. Various substances (particularly haem derivatives) enhance oxygen transport and have been shown to play an essential role in maintaining oxygen gradients, even in highly vascularized normal tissues with high aerobic and metabolic requirements *e.g.* muscle (Wittenberg, 1970). The possibility that the free blood which collects during growth of more rapidly growing haemorrhagic neoplasms in animals and man may not only facilitate oxygen transport to avascular zones of tumour but afford some oxygen storage capacity, may be relevant to a consideration of the oxygen effect in radiobiology and radiotherapy. Whilst anoxia may contribute to central necrosis which develops in rapidly growing tumours such as the Y-P388 and also in spontaneous anaplastic neoplasms, the defective supply of nutrients other than oxygen, and the accumulation of toxic metabolites, appear no less important to the production of necrosis. It is possible that small (radiobiologically significant) concentrations of oxygen could be maintained once necrosis occurs.

Previous similar studies of oxygen effect, based on a much less haemorrhagic but poorly vascularized Ehrlich tumour in mice, gave a lower OER value for radiation effect on the primary (OER = 1·5) for air breathing *versus* tourniquet occlusion and a marked increase (OER = 3·2) when the mouse was irradiated in hyperbaric oxygen at 45 p.s.i.g pressure (van den Brenk *et al.*, 1962). However, in these previous experiments tourniquet occlusion had increased radioresistance of normal tissues of the leg by a factor of only 1·7 and the corresponding OER for hyperbaric oxygen was 2·8. This suggests that under the conditions of the experiment both normal and tumour tissues of the mouse were inordinately anoxic, and therefore radioresistant, in air. This was probably due to the effects of anaesthesia causing respiratory depression and of limb traction reducing blood flow during irradiation. It is suggested that such tissue anoxia and radioresistance is produced more readily in the mouse than in a more robust animal such as the rat, but would be reversed by high pressure oxygen breathing.

Clearly experiments such as these, designed to elucidate the importance of oxygen effect on the response of tumours to irradiation, cannot be extrapolated to man. Not only does tumour vascularization appear to depend on rate of growth of tumour and its degree of differentiation, but the haemorrhagic factor may also be of importance. Furthermore, species differences exist with respect to the readiness with which normal tissues may be rendered radiobiologically hypoxic under experimental conditions. Considerable interest continues to be attached to the oxygen effect in tumour radiotherapy although there now seems to be more general agreement among clinicians that early tumours, for which local control rates are reasonably high under conventional conditions of radiotherapy, are probably adequately oxygenated "in air". In more advanced neoplastic disease, local control rates do decrease and some improvements have been obtained using high pressure oxygen, although its efficiency in reversing any tumour anoxia thought to be present has been questioned. However, such reservations cannot be attached to the tourniquet technique as used in the irradiation of sarcomata of the extremities in the human. Here, improvements obtained in results have been modest and do not come up to expectations. The question arises therefore whether large necrotic, and often haemorrhagic, tumours such as these are indeed radioresistant as a result of tissue anoxia, or for some other reason. That the presence of tumour necrosis causes radiobiologically significant anoxia to develop *ipso facto* is frequently taken for granted but appears to warrant further study.

### REFERENCES

ALPER, T. & HOWARD-FLANDERS, P. (1956) Role of Oxygen in Modifying the Radiosensitivity of *E. Coli. Nature, Lond.*, **178**, 978.

CLARK, E. R. & CLARK, E. L. (1932) Observations on the New Growth of Lymphatic Vessels as Seen in Transparent Chambers Introduced into the Rabbit's Ear. *Am. J. Anat.*, **51**, 49.

CLIFF, W. J. (1965) Kinetics of Wound Healing in Rabbit Ear Chambers, a Time Lapse Cinematographic Study. *Q. Jl exp. Physiol.*, **50**, 79.

FLOREY, LORD (1970) *General Pathology*, 4th Ed. London: Lloyd-Luke.

JAMIESON, D. & VAN DEN BRENK, H. A. S. (1963) Comparison of Oxygen Tensions in Normal Tissues and Yoshida Sarcoma of the Rat Breathing Air or Oxygen at 4 Atmospheres. *Br. J. Cancer*, **17**, 70.

NEEDHAM, A. E. (1952) *Regeneration and Wound Healing*. London: Methuen.

VAN DEN BRENK, H. A. S. (1956) Studies in Restorative Growth Processes in Mammalian Wound Healing. *Br. J. Surg.*, **43**, 525.

VAN DEN BRENK, H. A. S. (1959) The Effect of Ionizing Radiations on Capillary Sprouting and Vascular Remodelling in the Regenerating Repair Blastoma Observed in the Rabbit Ear Chamber. *Am. J. Roentg.*, **81**, 859.

VAN DEN BRENK, H. A. S., ELLIOTT, K. & HUTCHINGS, H. (1962) Effect of Single and Fractionated Doses of X-rays on Radiocurability of Solid Ehrlich Tumour and Tissue Reaction *in vivo* for Different Oxygen Tensions. *Br. J. Cancer*, **16**, 518.

VAN DEN BRENK, H. A. S., MADIGAN, J. P., KERR, R. A., CASS, N. M. & RICHTER, W. (1963) The Treatment of Malignant Disease of the Extremities by Megavoltage Irradiation under Tourniquet Anoxia. *J. Coll. Radiol. Aust.*, **7**, 142.

VAN DEN BRENK, H. A. S., MOORE, V. & SHARPINGTON, C. (1971) Growth of Metastases from P-388 Sarcoma in the Rat Following Wholebody Irradiation. *Br. J. Cancer*, **25**, 186.

VAN DEN BRENK, H. A. S. & SHARPINGTON, C. (1972) Effect of Local X-irradiation of a Primary Sarcoma in the Rat on Dissemination and Growth of Metastases: Dose-Response Characteristics. *Br. J. Cancer*, **25**, 812.

WITTENBERG, J. B. (1970) Myoglobin-Facilitated Oxygen Diffusion: Role of Myoglobin in Oxygen Entry into Muscle. *Physiol. Rev.*, **50**, 559.


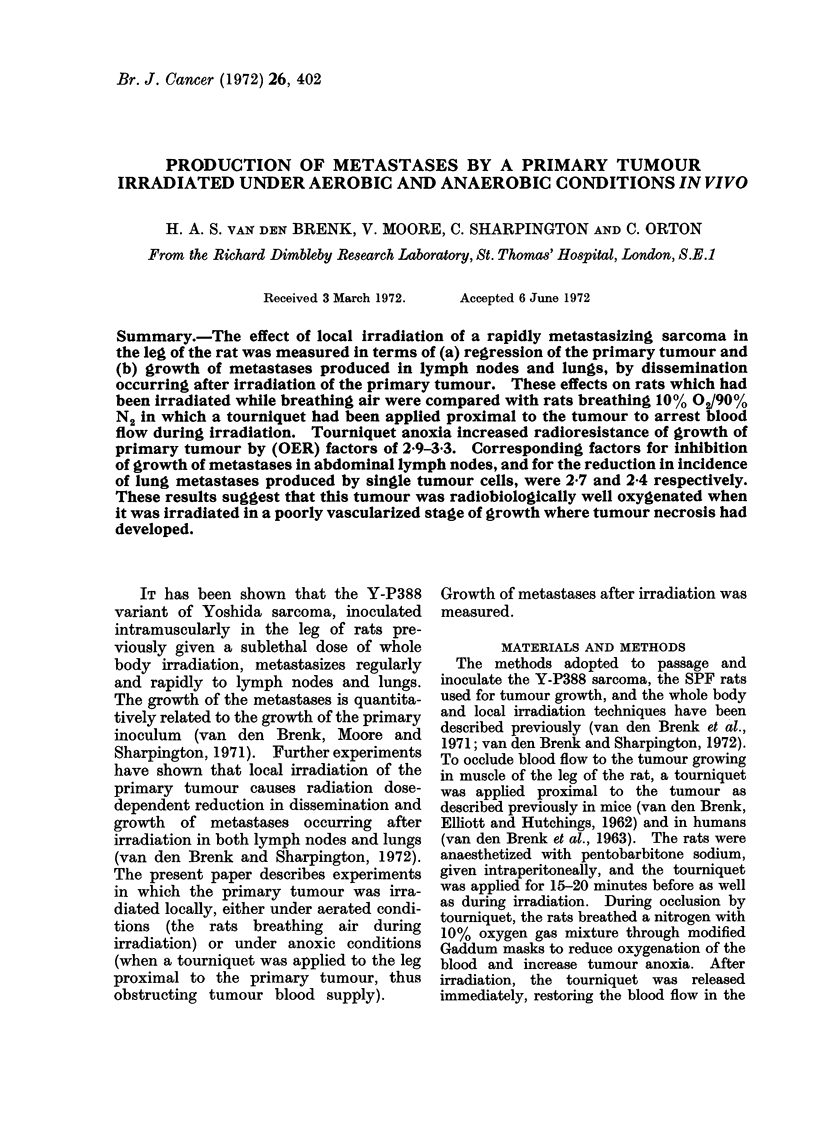

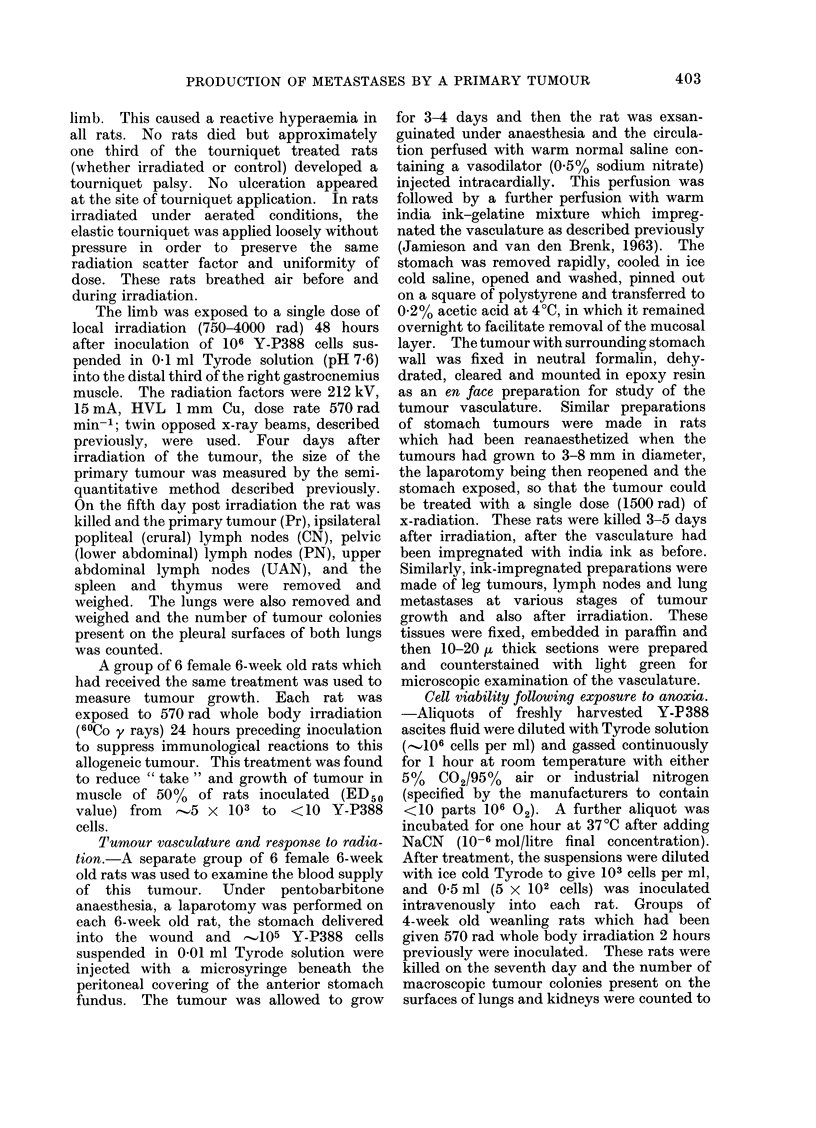

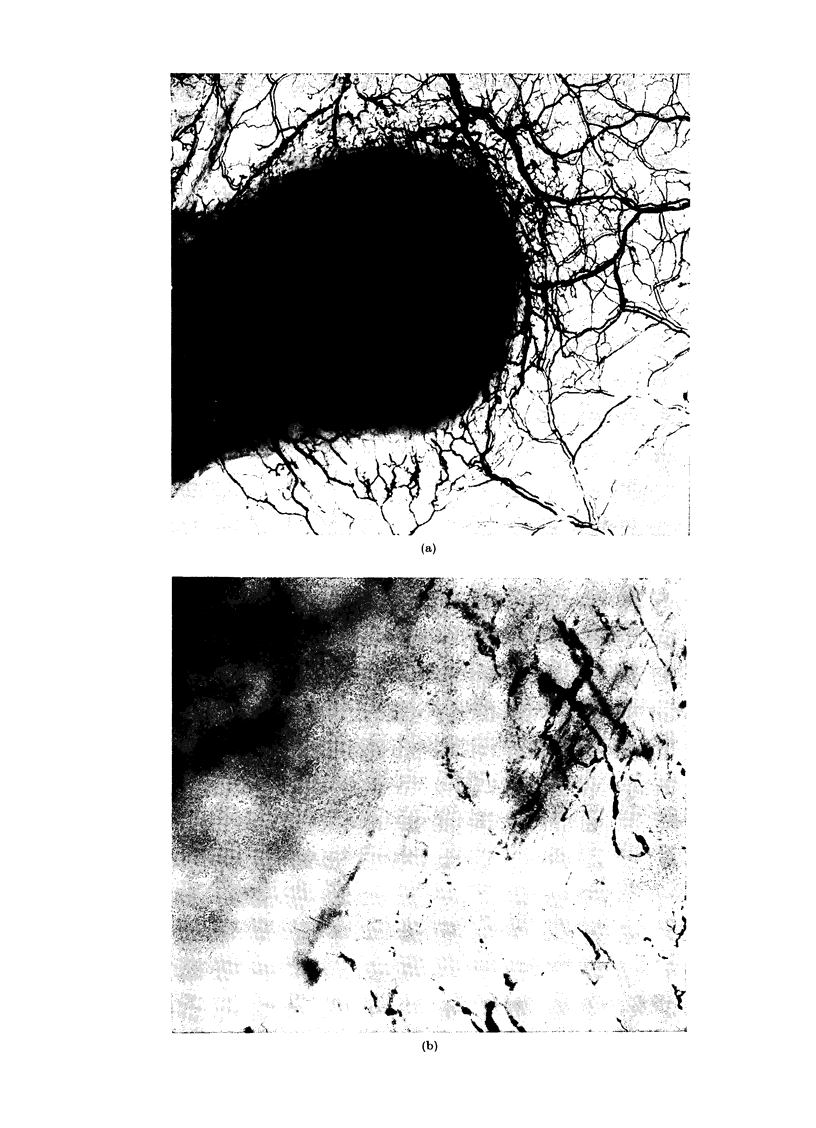

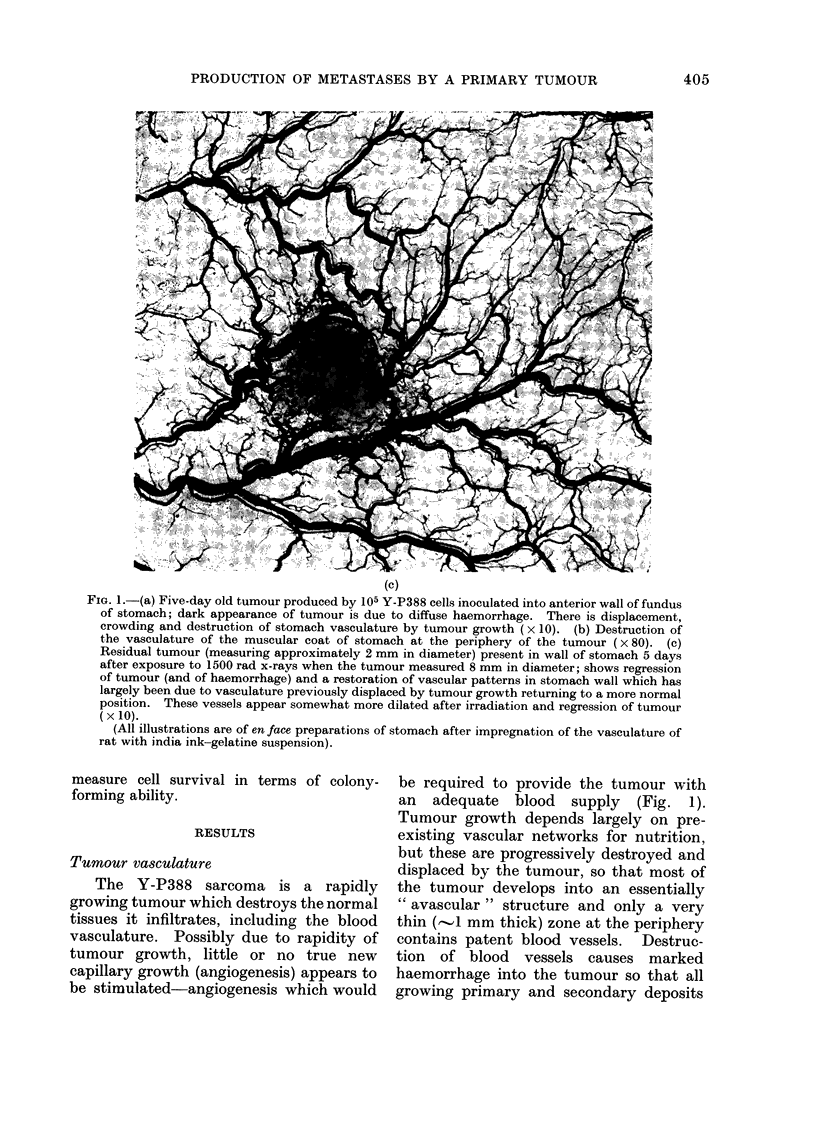

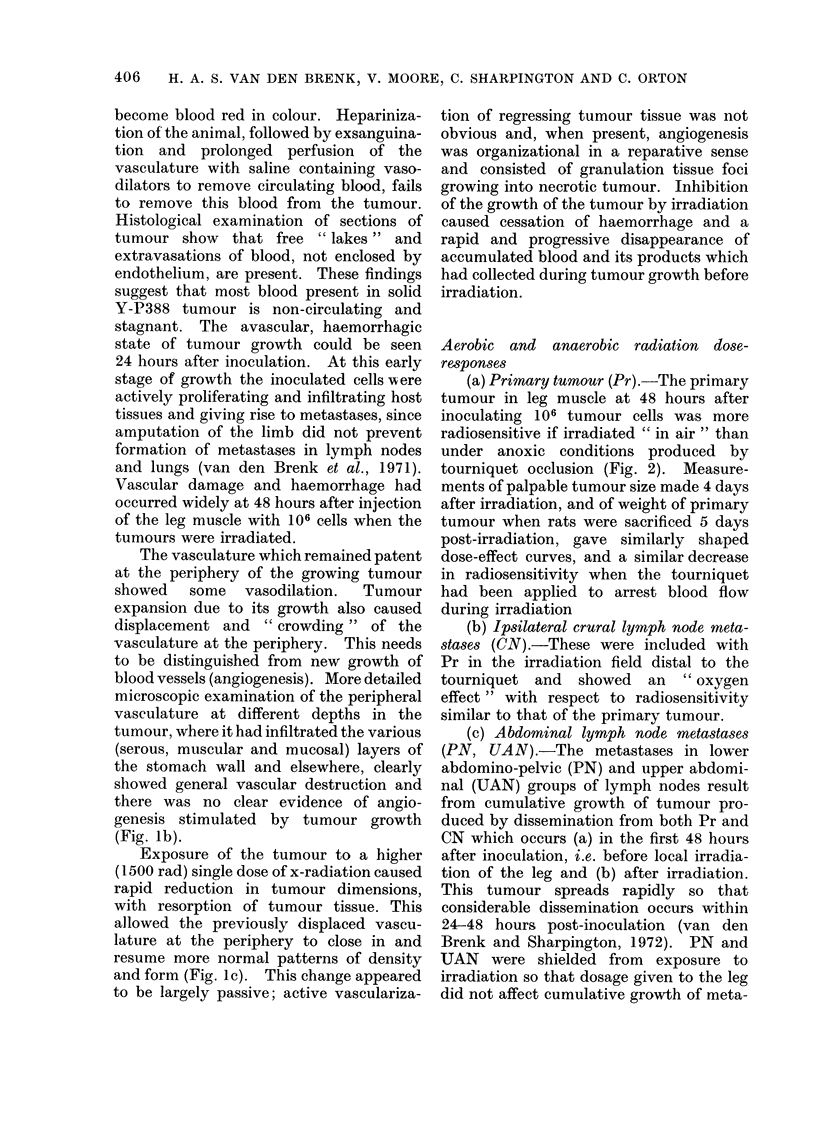

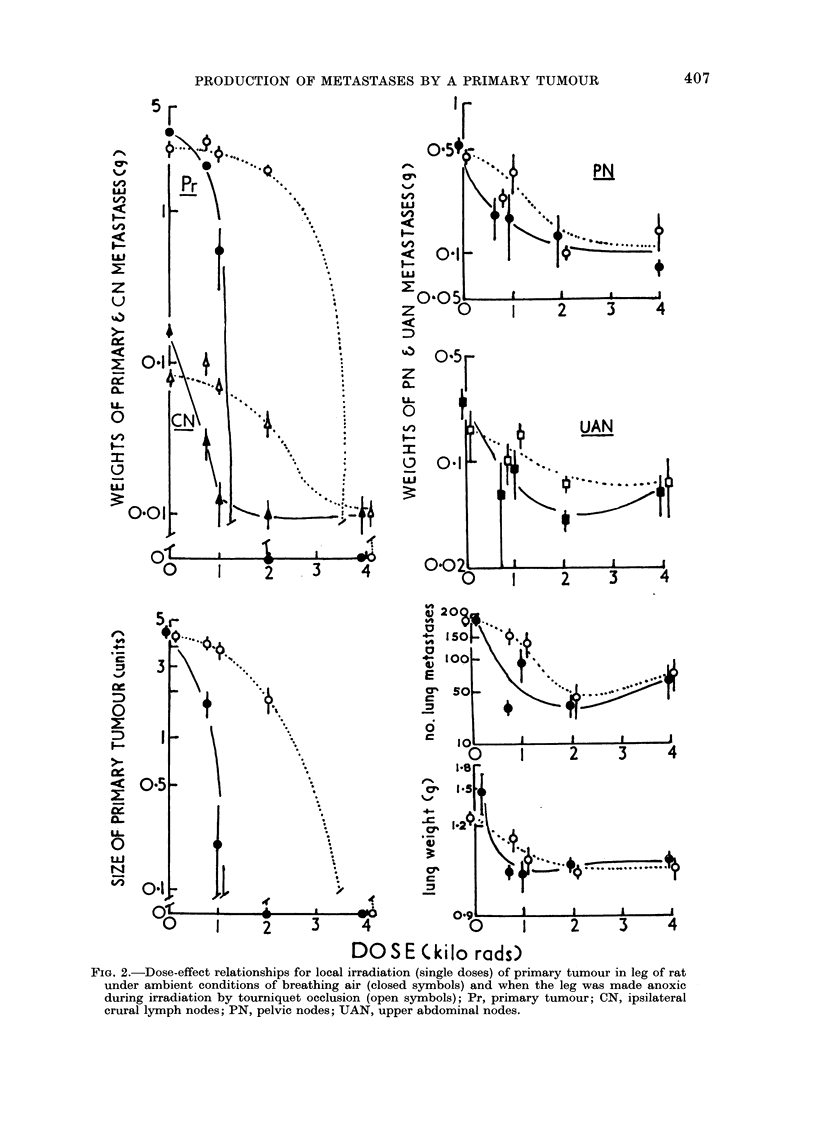

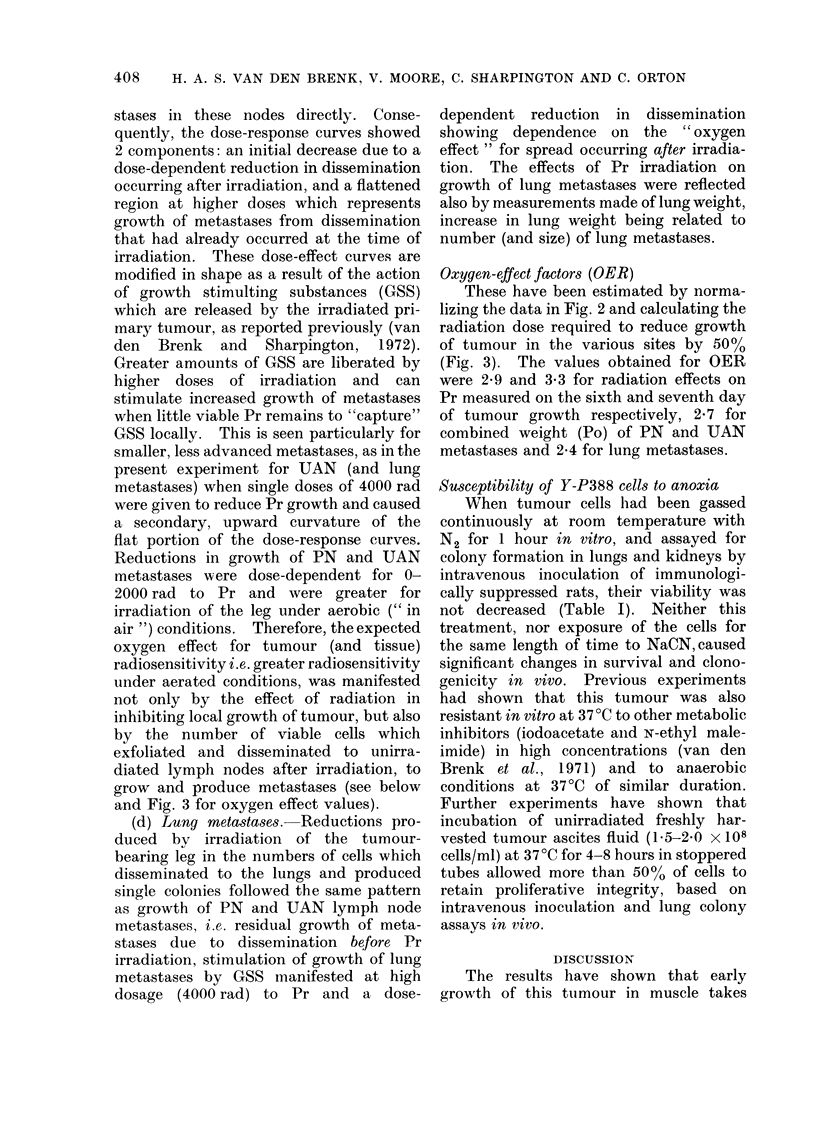

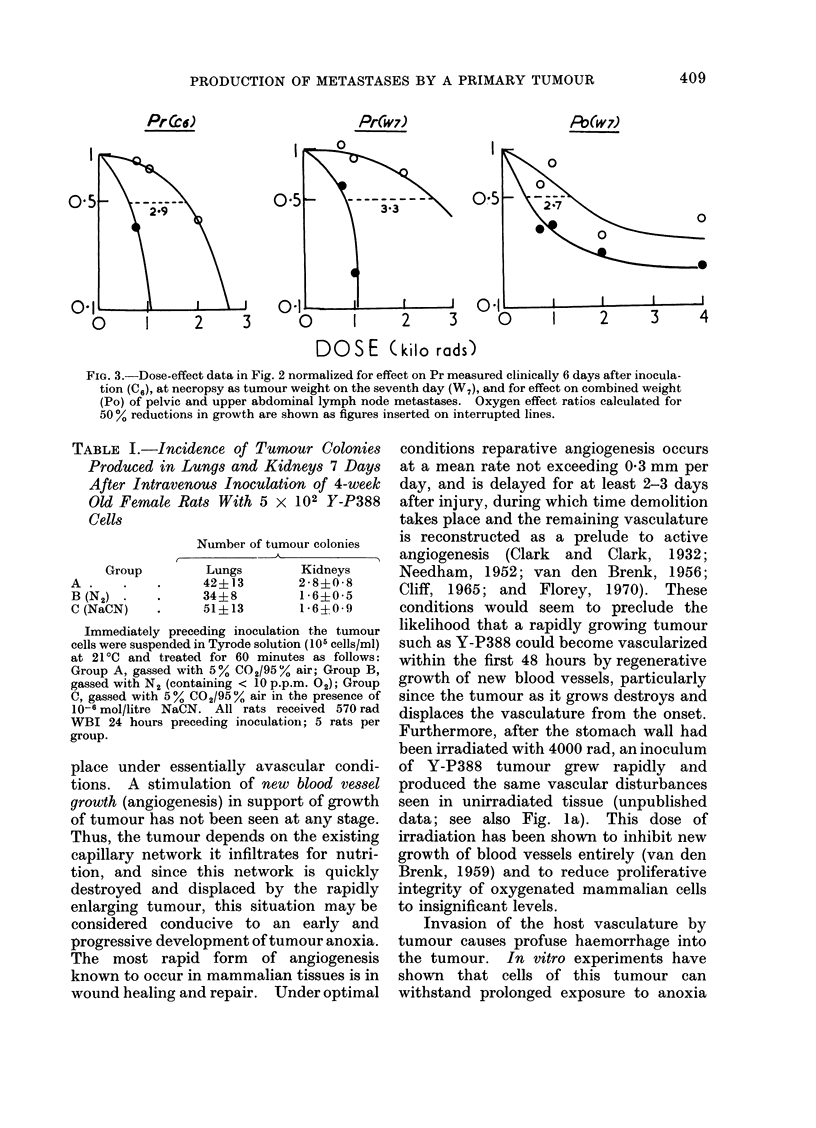

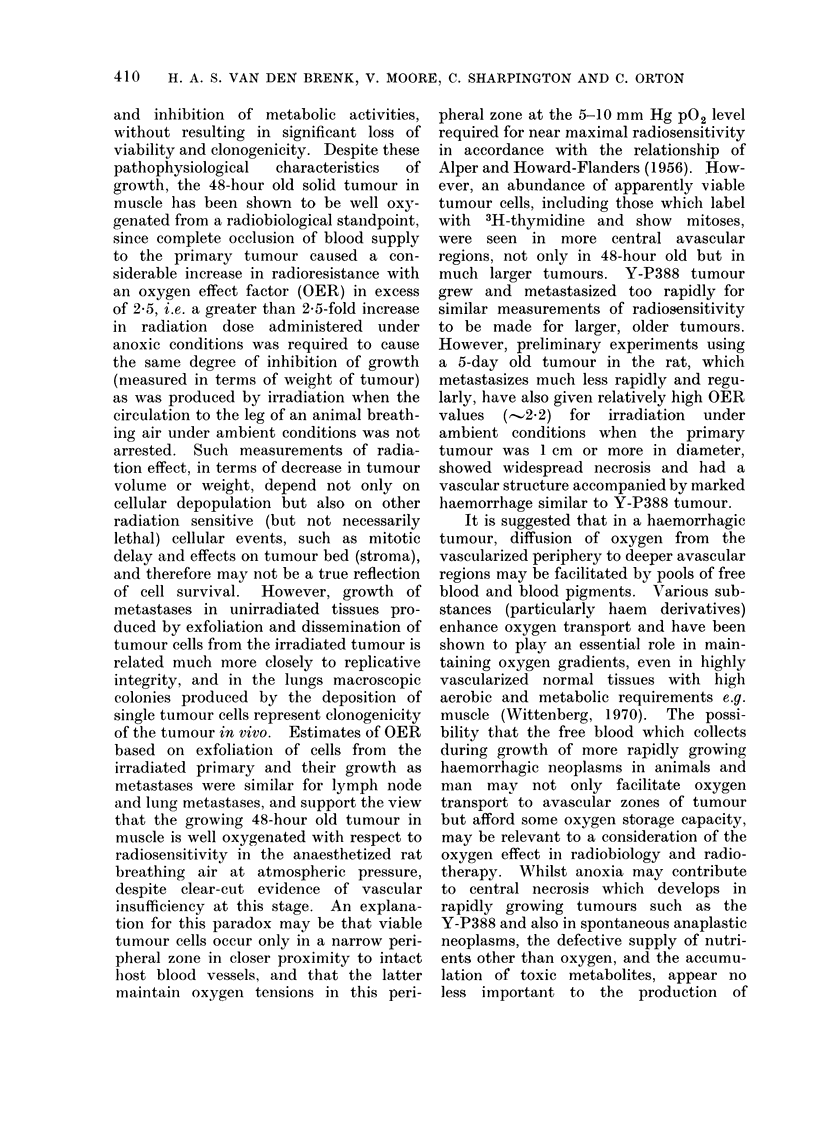

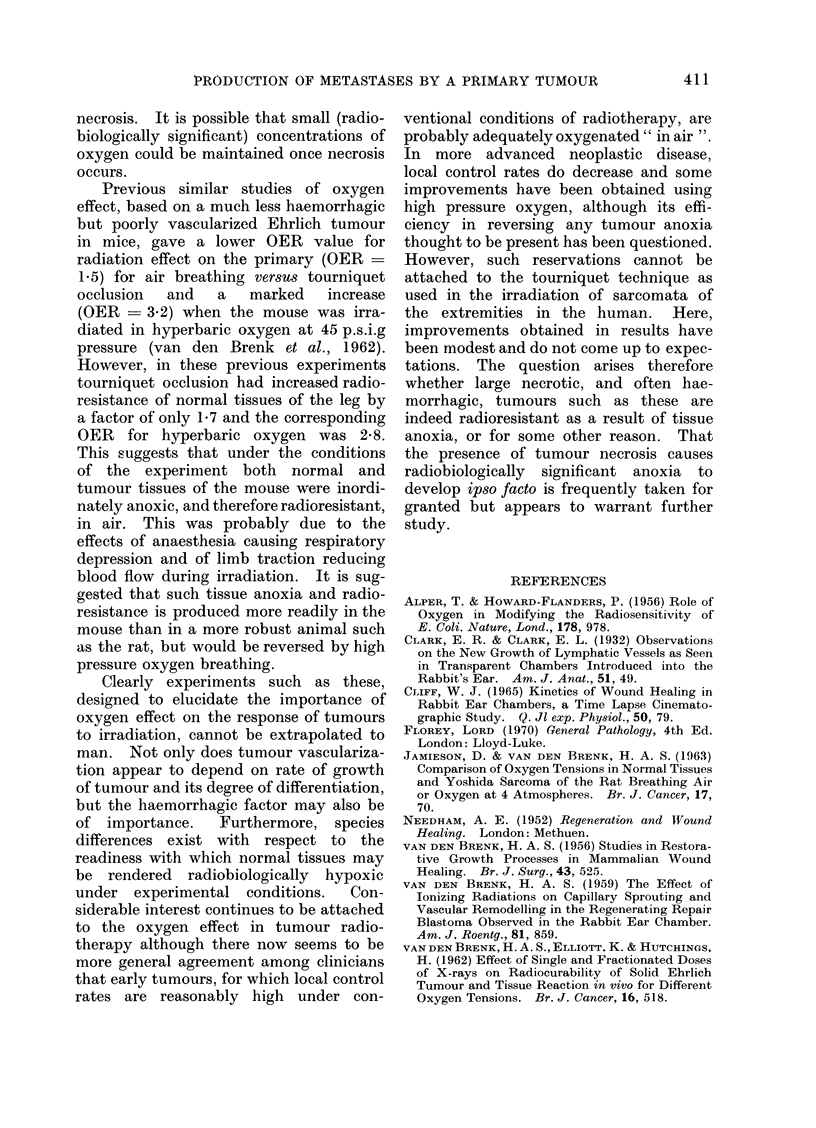

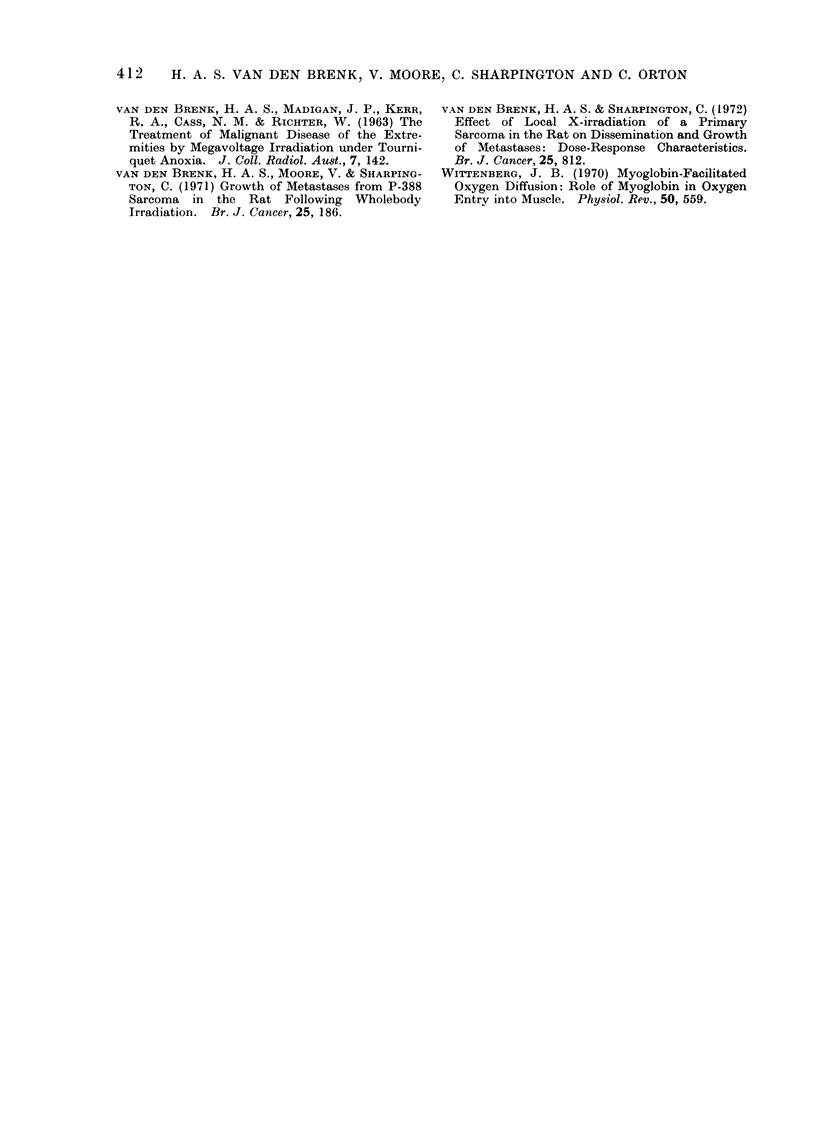


## References

[OCR_00964] ALPER T., HOWARD-FLANDERS P. (1956). Role of oxygen in modifying the radiosensitivity of E. coli B.. Nature.

[OCR_00975] CLIFF W. J. (1965). KINETICS OF WOUND HEALING IN RABBIT EAR CHAMBERS, A TIME LAPSE CINEMICROSCOPIC STUDY.. Q J Exp Physiol Cogn Med Sci.

[OCR_00984] JAMIESON D., VAN DEN BRENK H. A. (1963). Comparison of oxygen tensions in normal tissues and Yoshida sarcoma of the rat breathing air or oxygen at 4 atmospheres.. Br J Cancer.

[OCR_01000] VAN DEN BRENK H. A. (1959). The effect of ionizing radiations on capillary sprouting and vascular remodelling in the regenerating repair blastema observed in the rabbit ear chamber.. Am J Roentgenol Radium Ther Nucl Med.

[OCR_01016] VANDENBRENK H. A., MADIGAN J. P., KERR R. C., CASS N. M., RICHTER W. (1963). THE TREATMENT OF MALIGNANT DISEASE OF THE EXTREMITIES BY MEGAVOLTAGE IRRADIATION UNDER TOURNIQUET ANOXIA.. J Coll Radiol Australas.

[OCR_01025] Van den Brenk H. A., Moore V., Sharpington C. (1971). Growth of metastases from P-388 sarcoma in the rat followig whole body irradiation.. Br J Cancer.

[OCR_01029] Van den Brenk H. A., Sharpington C. (1971). Effect of local x-irradiation of a primary sarcoma in the rat on dissemination and growth of metastases: dose-response characteristics.. Br J Cancer.

[OCR_01036] Wittenberg J. B. (1970). Myoglobin-facilitated oxygen diffusion: role of myoglobin in oxygen entry into muscle.. Physiol Rev.

[OCR_01007] van den BRENK H., ELLIOTT K., HUTCHINGS H. (1962). Effect of single and fractionated doses of x-rays on radiocurability of solid Ehrlich tumour and tissue reactions in vivo, for different oxygen tensions.. Br J Cancer.

